# Thrombophilia and Immune-Related Genetic Markers in Long COVID

**DOI:** 10.3390/v15040885

**Published:** 2023-03-30

**Authors:** Rosilene da Silva, Kevin Matheus Lima de Sarges, Marcos Henrique Damasceno Cantanhede, Flávia Póvoa da Costa, Erika Ferreira dos Santos, Fabíola Brasil Barbosa Rodrigues, Maria de Nazaré do Socorro de Almeida Viana, Mauro de Meira Leite, Andréa Luciana Soares da Silva, Mioni Thieli Magalhães de Brito, Maria Karoliny da Silva Torres, Maria Alice Freitas Queiroz, Izaura Maria Vieira Cayres Vallinoto, Daniele Freitas Henriques, Carla Pinheiro dos Santos, Giselle Maria Rachid Viana, Juarez Antônio Simões Quaresma, Luiz Fábio Magno Falcão, Antonio Carlos Rosário Vallinoto, Eduardo José Melo dos Santos

**Affiliations:** 1 Laboratory of Genetics of Complex Diseases, Institute of Biological Sciences, Federal University of Pará, Belém 58255-000, Brazil; 2 Graduate Program in Biology of Infectious and Parasitic Agents, Federal University of Pará, Belém 58255-000, Brazil; 3 Graduate Program in Clinical Analysis, Federal University of Pará, Belém 58255-000, Brazil; 4 Laboratory of Virology, Institute of Biological Sciences, Federal University of Pará, Belém 58255-000, Brazil; 5 Section of Arbovirology and Hemorrhagic Fevers, Evandro Chagas Institute, Secretary of Health Surveillance, Ministry of Health of Brazil, Ananindeua 67000-000, Brazil; 6 Malaria Basic Research Laboratory, Parasitology Section, Evandro Chagas Institute, Health Surveillance Secretariat, Brazilian Ministry of Health, Ananindeua 67000-000, Brazil; 7 Center for Biological and Health Sciences, State University of Pará, Belém 58255-000, Brazil

**Keywords:** long COVID, risk factor, polymorphisms, *IFNG*, *MTHFR*

## Abstract

Aiming to evaluate the role of ten functional polymorphisms in long COVID, involved in major inflammatory, immune response and thrombophilia pathways, a cross-sectional sample composed of 199 long COVID (LC) patients and a cohort composed of 79 COVID-19 patients whose follow-up by over six months did not reveal any evidence of long COVID (NLC) were investigated to detect genetic susceptibility to long COVID. Ten functional polymorphisms located in thrombophilia-related and immune response genes were genotyped by real time PCR. In terms of clinical outcomes, LC patients presented higher prevalence of heart disease as preexistent comorbidity. In general, the proportions of symptoms in acute phase of the disease were higher among LC patients. The genotype AA of the interferon gamma (*IFNG*) gene was observed in higher frequency among LC patients (60%; *p* = 0.033). Moreover, the genotype CC of the methylenetetrahydrofolate reductase (*MTHFR*) gene was also more frequent among LC patients (49%; *p* = 0.045). Additionally, the frequencies of LC symptoms were higher among carriers of *IFNG* genotypes AA than among non-AA genotypes (Z = 5.08; *p* < 0.0001). Two polymorphisms were associated with LC in both inflammatory and thrombophilia pathways, thus reinforcing their role in LC. The higher frequencies of acute phase symptoms among LC and higher frequency of underlying comorbidities might suggest that acute disease severity and the triggering of preexisting condition may play a role in LC development.

## 1. Introduction

Since 2020, the coronavirus disease 2019 (COVID-19) constituted one of the greatest challenges in global public health, reaching over 600 million confirmed cases and a death toll of more than 6 million by the end of 2022. The most relevant pathogenesis pathways in the disease evolution are inflammatory cytokine storm and thrombophilia events [1,2,3,4].

Today, even after the vaccine development, COVID-19 is still presenting new chronic aspects of concern, such as the so-called long COVID (LC) that is not yet fully understood [5]. A number of acute COVID-19 patients developed prolonged or even new symptoms that could persist for over three months and maintained them for as long as 12 months or more [6]. According to the National Institute for Health and Care Excellence (NICE) from the United Kingdom [7], post-acute COVID-19 refers to persistence of symptoms over 3 weeks, but if the continuity of symptomatology reaches 3 months or more, it becomes chronic or long COVID. The proportion of long COVID showed to be heterogeneous, ranging from 31% in North Americans to 51% in Asians [8]. Moreover, the diagnosis of LC can be blurred by some factors, such as worsening of preexisting comorbidities and even post-intensive care syndrome in severe COVID-19 cases [9,10].

There are several obscure issues in LC, such as the prevalence and prognostic/predictive factors. While for acute COVID-19, severity-associated factors such as age, sex and comorbidities were immediately recognized, for LC, the risk factors are still poorly known. Some studies point to a putative association with cytokine levels, *IL-2, IL-4, IL-10* and *IL-17* [11]. However, the role of genetic factors has been poorly approached and no significant genetic associations have been reported [12].

The present study aimed to evaluate the role of ten functional polymorphisms at ten genes coding for cytokines involved in major inflammatory pathways of COVID-19 and proteins associated with risk of thrombophilia, as well as other immune relevant SNPs associated with regulation of class II HLA and dendritic cell receptor expression.

## 2. Materials and Methods

### 2.1. Study Design and Ethic Aspects

The present study was composed of two sample groups, a cross-sectional group composed of 199 long COVID patients and a cohort composed of 79 COVID-19 patients whose follow-up by over six months did not reveal any evidence of long COVID. Both sample groups were selected from a larger sample according to very rigorous inclusion/exclusion criteria, as described below.

All patients of both sample groups had their diagnosis of COVID-19 confirmed by RT-PCR, with clinical symptoms and recovery information obtained from medical records. The same clinical parameters and health multiprofessional approaches were used to evaluate and classify all patients. The sampling was performed between July 2020 and December 2021 and included patients that are residents in Belém (Pará, Brazil) from both sexes, over 18 years old and unvaccinated during the time of the study. Additionally, no reinfection could be detected or reported among both sample groups. The severity of acute COVID-19 was evaluated according to WHO criteria [13], from information on medical records.

The non-long COVID patients (NLC) constituted of 76 patients that had mild acute COVID-19, with no need for hospitalization or supplemental oxygen, and three patients that were asymptomatic, but had SARS-CoV-2 infection confirmed by real time PCR. The exclusion of severe and moderate COVID-19 patients was conducted in order to match more properly this sample with the long COVID sample. Only patients whose medical records and mutiprofessional follow-up allowed to exclude any new signal or symptom that could be assigned to long COVID development were included. 

The long COVID sample (LC) was constituted of patients screened by the Comprehensive Health Care Program for Patients with long COVID of State University of Pará. Among the patients obtained using this service, 199 patients were screened based on the following criteria: (i) reporting long COVID symptoms and sequalae by over three months after the acute infection, the symptoms and sequelae were evaluated and confirmed by a multiprofessional team composed of physiotherapists and specialized physicians, and by image and laboratory exams; (ii) only patients with SARS-CoV-2 RNA detection by RT-PCR and complementary exams were accepted in the sample; (iii) Only patients that presented mild acute COVID-19 were included in the sample in order to avoid confounding sequelae, as those from intensive care syndrome with long COVID signs and symptoms [9].

Personal, demographic and clinical data were collected using cryptographed Google forms™, stored in computers with controlled access by individual passwords.

The present study was approved by the National Ethic Committee (CAEE: 33470020.1001.0018; protocol number nº 2.190.330). All the participants provided written informed consent. This study was conducted in strict accordance with the principles of the Declaration of Helsinki and followed recommendations provided by the guidelines for reporting observational studies, the STrengthening the REporting of Genetic Association studies (STREGA) [14].

Variables used for describing and subgrouping of the final sample were age, sex, main symptoms presented during the acute phase of COVID-19 and LC, duration of symptoms (LC) and severity of the acute phase of COVID-19.

### 2.2. Sample Processing and Genotyping

DNA was isolated from venous blood samples (4 mL) and collected using EDTA as the anticoagulant. DNA isolation was performed using the kit ReliaPrepTM Blood gDNA Miniprep System (Promega), following the protocol recommended by the fabricant.

Ten SNPs were chosen based on their functional characteristics, most of them related to modulation of gene expression, localized in cytokine loci *IFNG* (+874T/A; rs2430561), *TNFA* (-308G/A; rs1800629), *IL6* (-174G/C; rs1800795) and *IL6R* (358A/C; rs2228145); thrombophilia associated loci *MTHFR* (C677T, Ala222Val; rs1801133) and *FV* Leiden (R506Q C/T; rs6025); putative SARS-CoV-2 receptor [15] and antigen presenting cell receptor *CD209* (-336A/G; rs4804803); and expression regulator polymorphisms of Class II HLA loci *CIITA* (-168A/G; rs3087456), *HLA-DPA1* (rs3077) and *HLA-DPB1* (rs9277534).

The SNPs genotyping was performed by real time PCR using pre-designed assays (Thermo Fisher, Carlsbad, CA, EUA) in a sequence detector StepOne PLUS (Applied Biosystems, Foster City, CA, EUA), following the fabricant protocols. The assays ID are as follows: *TNFA* (C___7514879_10); *IL6* (C___1839697_20); *IL6R* (C__16170664_10); *MTHFR* (C___1202883_20); *FV* Leiden (C__11975250_10); *CD209* (C___1999340_10); *CIITA* (C__15793789_10); *HLA-DPA1* (C__11916951_10); *HLA-DPB1* (C__29841700_20). The *IFNG* (+874T/A; rs2430561) real time genotyping protocol was previously described [16].

### 2.3. Statistical Analyses

All SNPs were tested for Hardy-Weinberg equilibrium. The genotype and allele frequencies of each SNP were estimated by direct count. Comparison of the allele and genotype frequencies between LC and NLC groups were carried out using Fisher exact test. The comparison of the frequencies of symptoms between different genotype carriers were performed using paired Wilcoxon test.

For six SNPs, genotype and allele frequencies in the population of Belém could be obtained from studies conducted before the COVID-19 pandemics [17,18,19,20,21,22,23,24], as detailed in Supplementary Table S1 (Supplementary Materials), hence, representing the frequencies in the general population of Belém without any sampling biases putatively induced by the pandemics.

Correction for multiple tests is usually made in genes with several alleles because there is only one hypothesis to be tested with many tests. Otherwise, multiple genes represent one hypothesis per gene. However, some studies apply corrections across multiple genes, such as GWAS. The crossroad in this issue is how conservative the authors want to be in their conclusions. Thus, we opted to present the raw test results without correction, allowing the readers to take their own conclusions.

## 3. Results

Demographic and clinical characteristics of the samples are presented in Table 1. Female predominates in both samples, as well as ages under 60 years, and the proportion of females is higher among LC patients than in the general population. In terms of clinical characteristics, LC presented higher prevalence of heart disease as a preexisting comorbidity (Fisher exact test; *p* = 0.001), and the proportions of the remaining comorbidities were similar to those observed in the NLC sample. Moreover, in general the proportions of symptoms in acute phase of the disease were higher among LC patients (Wilcoxon test; Z = 2.9; *p* = 0.0032), fatigue being by far the most frequent, (observed in 53% of the patients), followed by anosmia/hyposmia/parosmia (29%).

The exclusion of asymptomatic patients did not change the significance of the statistical significance observed.

**Figure 1:**
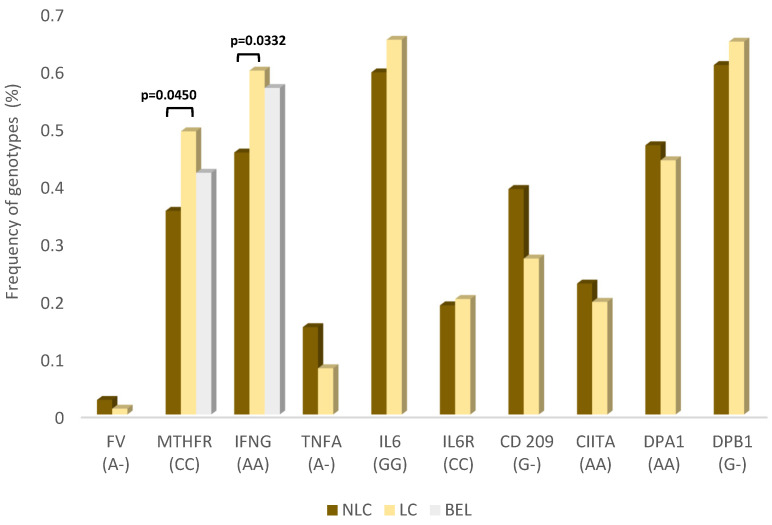
Frequency (%) of single nucleotide polymorphism (SNP) genotypes in patients with non-long COVID (NLC; N = 79), long COVID (LC; N = 199) and in the general population of Belém (Bel; obtained from published papers prior to the pandemics, thus representing the frequencies in general population of Belém without any sampling biases induced by the pandemics). NLC are patients who have not reported symptoms or sequelae after COVID-19. LC are patients with reported symptoms for over 3 months. The SNPs are discriminated information about genotypes and their respective effects in gene product expression are given. For LC and NLC subsamples composed of asymptomatic or mild or patients. Fisher’s exact test.

Complete genotype and allele frequencies of all ten SNPs, in both LC and NLC groups, are presented in Supplementary Table S1. Two SNPs, rs2430561 (Interferon Gamma, *IFNG*) and rs1801133 (Methylenetetrahydrofolate Reductase, *MTHFR*), showed statistical differences between LC and NLC groups, as highlighted in Figure 1. Even the exclusion of the asymptomatic patients did not alter these results.

The genotype AA of *IFNG* gene, associated with lower expression, was observed in higher frequency among long COVID patients (60%), and this difference was statistically significant (Fisher Exact test; *p* = 0.033).

Moreover, the genotype CC of *MTHFR* gene, associated with higher expression, was also more frequent among long COVID patients (49%). Fisher Exact test showed significance (*p* = 0.045).

The genotypes AA of *IFNG* and CC of *MTHFR* in the population of Belém, according to data gathered from studies conducted prior the COVID-19 pandemics, showed frequencies of 56.8% and 42.1%, respectively (Figure 1).

**Figure 2:**
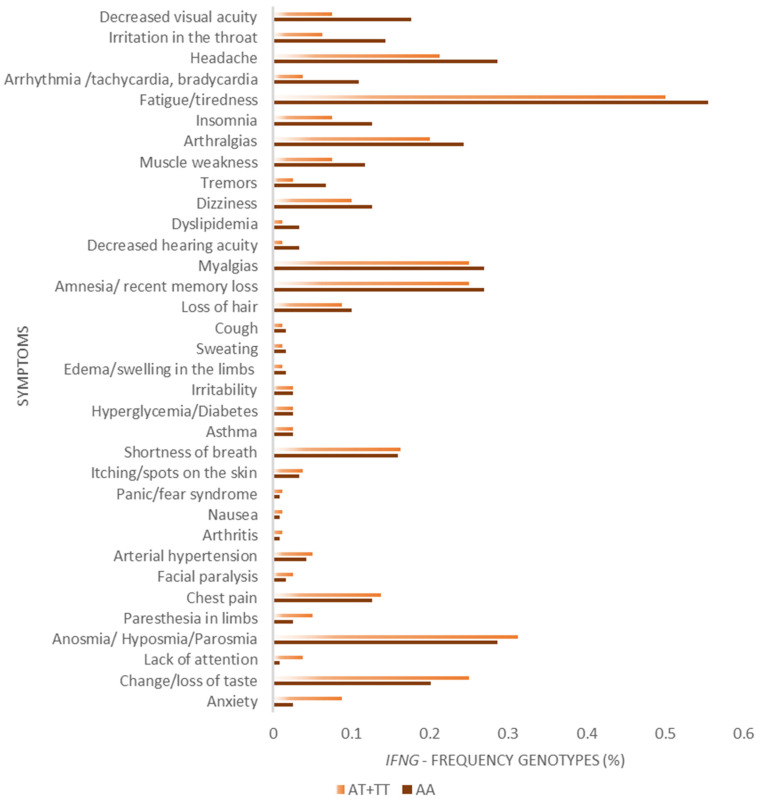
Comparison of frequencies of the main symptoms (%) during long COVID among *IFNG* AA and AT+TT genotype carriers (Paired Wilcoxon test; Z = 5.08; *p* < 0.0001).

**Figure 3:**
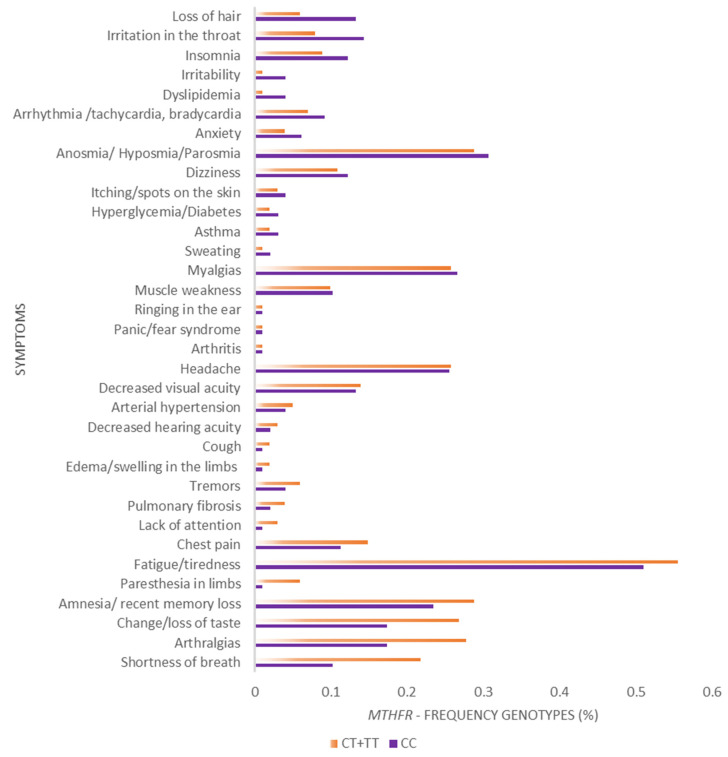
Comparison of Frequencies of the main symptoms (%) during long COVID among *MTHFR* CC and CT+TT genotype carriers (Paired Wilcoxon test: Z = 0.31, *p* = 0.7489).

Additionally, the frequencies of long COVID symptoms between carriers of *IFNG* genotypes AA and non-AA were performed across all symptoms by paired Wilcoxon test (Figure 2), showing a strong statistical significance in higher frequencies of symptoms among AA carriers (Z = 5.08; *p* < 0.0001). However, the frequencies of symptoms were not statistically different in *MTHFR* CC genotypes compared with non-CC ones (Figure 3).

## 4. Discussion

The female gender was more frequent among LC patients than among NLC ones. This result is in agreement with previous studies that suggested an association of long COVID with female gender [25,26,27,28]. Additionally, our results also showed the incidence of LC among patients younger than 60 years, which was also reported in previous studies [29,30].

Cardiac disease was the most prevalent preexistent comorbidity among LC compared to NLC patients. Some studies showed that preexisting comorbidities might potentialize or increase the risk of prolonged symptoms associated to long COVID [13,29]. In this context, there is evidence that COVID-19 patients show an augmented risk of cardiovascular disorders, even in the absence of previous heart disease [31], providing a link between cardiovascular disease development and long COVID.

The frequencies of symptoms during COVID-19 acute phase were clearly higher in the LC group. These results could represent a putative association with COVID-19 severity, in agreement with previous studies that associated the severity of the acute phase with persistent symptoms [2].

An additional point is that cardiovascular disease has been widely associated to the allele T of the rs1801133 from *MTHFR* loci. Thus, the higher frequency cardiac disease among LC patients could not be a consequence of the genotype CC’s higher frequency in this group [32].

Polymorphisms of *MTHFR* gene have been reported in association with several diseases, including cardiovascular diseases, thrombophilia predisposition, inflammatory disorders and even cancer [32]. Regarding infectious diseases, mutations at this gene could be associated with important protozoa infections such as malaria and leishmaniosis [33,34], as well as with viral diseases such as human papilloma virus [35] Cytomegalovirus, HIV and Crimean-Congo hemorrhagic fever [36,37,38]. While most of the studies indicate predisposition due to the presence of *MTHFR* *T allele, at least one study reported protective effects of this allele against persistent HBV infection in West Africa [39].

The associations of this polymorphism with COVID-19 severity have been suggested by meta-analysis based on the correlation of T allele frequencies with COVID-19 mortality [40]. However, to this date, no studies have been conducted investigating the role of *MTHFR* mutations in long COVID. Our results are the first to provide initial clues on the relationship of impaired folate-mediated one carbon metabolism with long COVID.

However, carriers of the *MTHFR* predisposing genotype do not show differences in the long COVID symptoms frequencies, if compared to non-carriers. This result, along with a low significance of p-value of the Fisher exact test, can be indicative of a spurious association.

After SARS-CoV-2 infection, a persistent inflammatory response could be detected for about 40–60 days, even among patients with mild and asymptomatic COVID-19 [3]. In this context, long COVID is assumed to be related to residual inflammation and tissue damage in association with preexisting comorbidities [41]. Indeed, a previous study from our group suggested a molecular signature of Th17 inflammatory profile with a decrease in IL-4 and IL-10 anti-inflammatory cytokine levels [11].

Following a simple logic, the SNP associated with a low expression of *IFNG* would lead to low plasma levels of IFN-γ. If this polymorphism is associated with long COVID, it would be expected that in our previous paper [11], low levels of IFN-γ should be detected in plasma, but this was not the case. However, the present study used only LC patients that had mild acute COVID-19, while the previous cytokine paper also used patients that had severe acute COVID-19 patients. Thus, they are not directly comparable. Moreover, during the development of Th17 immune response pattern the dynamics of interferon-γ production is not linear. It is known that in some situations, interferon-gamma can negatively regulate Th17-mediated immunopathology [42]. Thus, low *IFNG* expression can be associated with initial Th17 profile development. Moreover, pathogen-induced Th17 cells are also able to produce IFN-γ afterwards [43]. In this scenario, during an infection followed by Th17 profile establishment, early and late IFN-γ plasma levels would not necessary be similar.

In conclusion, the association of long COVID with interferon gamma gene polymorphism seems to be a valuable clue for understanding the underlying mechanisms. Not only was the frequency of the low expression CC genotype associated with long COVID, but the long COVID symptoms showed to be more frequent among CC genotype carriers when compared with the remaining genotypes. Thus, due to the high significance, even after correction for multiple tests, we considered that the rs2430561, at the *INFG* gene, can be an important direct or indirect marker for long COVID.

Interferon gamma is a key factor in viral infections, involved in several immunological pathways, such as antigen processing and presentation, apoptosis, antiviral mediators, lysosome mediated killing/phagosome maturation and complement pathway, among others [44]. Thus, genotypes that modulate INF-γ expression could influence the persistence of COVID-19 symptomatology.

Despite the scarcity of studies in long COVID host genetics, some aspects of *IFNG* reveals interesting links between inflammatory pathways related to long COVID. Indeed, INF-γ was identified as an important mediator in controlling sortilin-1 (Sort-1) levels, which is a receptor of VPS10p family associated with cardiovascular disease [45], including the reduction in Sort-1 by INF-γ regulated by JAK/STAT pathway.

Interestingly, Sort-1 is associated with several diseases, including inflammation syndromes [46]. Thus, INF-γ low expression genotype could display lower *Sort-1* inhibition, predisposing to inflammatory profiles underlying long COVID pathogenesis.

Moreover, by analogy with other viral infections presenting long lasting inflammatory/immune-based diseases, retroviral chronic infections showed higher INF-γ levels associated with inflammatory symptoms [47,48]. However, in such diseases, the viral infection persists along with the inflammatory symptomatology. Alternatively, some viral infections, such as hemorrhagic fevers, could present immune-based disease after the viral infection clearance. In this context, a gene expression study highlighted the role of INF-γ in the protection against hemorrhagic dengue fever [49], in agreement with our results, suggesting that low expression genotypes are predisposed to long COVID and the higher expression genotypes are protective.

Despite presenting promising genetic associations, our study has limitations and strengths, such as the need for future studies with larger samples and rigorous follow-up and controlling of long COVID patients in order to evaluate long COVID evolution patterns and their putative host genetics basis. The value of our results resides in the first multigenic approach with rational choice of candidate polymorphisms in well delimited samples for absence or occurrence of long COVID, controlled for acute disease severity. Even with the limitation of sample size, it was possible to detect associations that will guide future studies.

## 5. Conclusions

The present study identified, among ten candidate genes, two polymorphisms associated with long COVID in both inflammatory and thrombophilia major pathways. The genetic basis of long COVID triggering is scarce and our study is among the first ones to approach the underlying host genetic factors of the disease. The results provide valuable clues for future studies, such as homocysteine plasma levels evaluation, and begin to reveal the extensive complexity of the long-lasting symptomatology of COVID-19.

## Figures and Tables

**Table 1:** Demographic and clinical characteristics of long COVID and non-long COVID samples.

Variables	NLC * n = 79 (28%)	LC * n = 199 (72%)	Total *n* = 278 (100%)
**Sex (*n*, %)**			
Female	40 (51%)	140 (70%)	180 (65%)
Male	39 (49%)	59 (30%)	98 (35%)
**Age range (years) (*n*, %)**			
≤59	76 (96%)	169 (85%)	245 (88%)
≥60	3 (4%)	30 (15%)	33 (12%)
Age median (years)	43 ± 14	48 ± 13	46 ± 14
**Smoker or ex-smoker**			
Yes	4 (5%)	16 (8%)	20 (7%)
No	75 (95%)	183 (92%)	258 (93%)
**Major Comorbidities**			
Heart Diseas.	6 (7%) ^a^	48 (24%) ^a^	54 (19%)
Obesity	6 (7%)	12 (6%)	18 (6.5%)
Asthma	0	17 (8.5%)	17 (6.1%)
Diabetes Mellitus	0	7 (3.5%)	7 (2.5%)
Arterial hypertension	2 (2%)	4 (2%)	6 (2%)
Liver Disease	0	4 (2%)	4 (1.4%)
Immunosuppression, autoimmune disease	0	3 (1.5%)	3 (1.0%)
Chronic kidney disease	1 (1.3%)	1 (0.5%)	2 (0.7%)
Hypothyroidism	1 (1.3%)	1 (0.5%)	2 (0.7%)
**Major symptoms—acute phase COVID-19 (*n*, %)**			
Myalgias	41 (52%)	155 (78%)	196 (70%)
Headache	49 (63%)	140 (70%)	189 (68%)
Loss of taste	46 (58%)	137 (69%)	183 (66%)
Fatigue/tiredness	36 (45%)	146 (73%)	182 (65%)
Anosmia	48 (61%)	127 (64%)	175 (63%)
Fever	49 (62%)	125 (63%)	174 (62%)
Cough	40 (51%)	117 (59%)	157 (56%)
Shortness of breath	23 (29%)	123 (62%)	146 (52%)
Muscle weakness	37 (47%)	109 (55%)	146 (52%)
Chest pain	26 (33%)	113 (57%)	139 (50%)
Sore throat	32 (40%)	106 (53%)	138 (49%)
Diarrhea	27 (34%)	76 (38%)	103 (37%)
Coryza	29 (37%)	71 (35%)	100 (36%)
Abdominal pain	14 (17%)	65 (33%)	79 (28%)
Nausea	11 (14%)	64 (32%)	75 (27%)
Retro-orbital pain	24 (30%)	36 (18%)	60 (22%)
Vomit	6 (7%)	42 (21%)	48 (17%)
Disease severity—acute phase COVID-19 (*n*, %) **			
Asymptomatic	3 (4%)	0	3 (1%)
Mild	76 (96%)	199 (100%)	275 (99%)
**Number of symptoms presented in long COVID (*n*, %)**			
Up to 3 symptoms	-	121 (61%)	-
More than 3 to 9 symptoms	-	67 (34%)	-
More than 9 to 15 symptoms	-	11 (5%)	-
**Major symptoms in long COVID (*n*, %)**			
Fatigue/tiredness	-	106 (53%)	-
Anosmia/hyposmia/parosmia	-	59 (29%)	-
Myalgias	-	52 (26%)	-
Amnesia/ recent memory loss	-	52 (26%)	-
Headache	-	51 (25%)	-
Arthralgias	-	45 (22%)	-
Change/loss of taste	-	44 (22%)	-
Shortness of breath	-	32 (16%)	-
Decreased visual acuity	-	27 (13%)	-
Chest pain	-	26 (13%)	-
Dizziness	-	23 (11%)	-
Irritation in the throat/throat	-	22 (11%)	-
Insomnia	-	21 (10%)	-
Muscle weakness	-	20 (10%)	-
Loss of hair	-	19 (9%)	-
Arrhythmia	-	16 (8%)	-
Anxiety	-	10 (5%)	-
Tremors	-	10 (5%)	-
Arterial hypertension	-	9 (4%)	-
Itching/spots on the skin	-	7 (3%)	-
Paresthesia in limbs	-	7 (3%)	-
Pulmonary fibrosis	-	6 (3%)	-
Asthma	-	5 (2%)	-
Decreased hearing acuity	-	5 (2%)	-
Dyslipidemia	-	5 (2%)	-
Hyperglycemia/Diabetes	-	5 (2%)	-
Irritability	-	5 (2%)	-
Facial paralysis	-	4 (2%)	-
Lack of attention	-	4 (2%)	-
Edema/swelling in the limbs	-	3 (1.5%)	-
Sweating	-	3 (1.5%)	-
Arthritis	-	2 (1%)	-
Depression	-	2 (1%)	-
Nausea	-	2 (1%)	-
Panic/fear syndrome	-	2 (1%)	-
Ringing in the ear	-	2 (1%)	-
Weight gain/loss	-	2 (1%)	-
Urinary incontinence/dysuria	-	1 (0.5%)	-

* LC—Long COVID at the time of evaluation (>3 months); NLC—non-long COVID. ** Classified according to criteria established by the World Health Organization (WHO). ^a^ Significant difference (Fisher exact test; *p* = 0.001).

## Data Availability

The raw data supporting the conclusions of this article will be made available by the authors without undue reservation.

## Supplementary Materials

The following supporting information can be downloaded at: https://www.mdpi.com/article/10.3390/v15040885/s1, Table S1. Genotypic and allelic frequencies of genetic markers in relation to acute COVID-19 (NLC) patients, long COVID patients (LC) and the general population in Belém (BEL; prior the pandemics).

## References

[1] Marco Cascella A., Rajnik M., Cuomo A., Dulebohn S.C., di Napoli R. (2020). Italy Uniformed Services Un of the Health Sc Istituto Nazionale Tumori-IRCCS-Fondazione Pascale. https://pubmed.ncbi.nlm.nih.gov/32150360/.

[2] Huang C., Huang L., Wang Y., Li X., Ren L., Gu X., Kang L., Guo L., Liu M., Zhou X. (2021). 6-Month Consequences of COVID-19 in Patients Discharged from Hospital: A Cohort Study. Lancet.

[3] Doykov I., Hällqvist J., Gilmour K.C., Grandjean L., Mills K., Heywood W.E. (2020). “The Long Tail of COVID-19”—The Detection of a Prolonged Inflammatory Response after a SARS-CoV-2 Infection in Asymptomatic and Mildly Affected Patients. F1000Research.

[4] del Rio C., Collins L.F., Malani P. (2020). Long-Term Health Consequences of COVID-19. JAMA—J. Am. Med. Assoc..

[5] Mehandru S., Merad M. (2022). Pathological Sequelae of Long-Haul COVID. Nat. Immunol..

[6] Greenhalgh T., Knight M., A’Court C., Buxton M., Husain L. (2020). Management of Post-Acute COVID-19 in Primary Care. BMJ.

[7] (2020). National Institute for Health and Care Excellence (NICE) COVID-19 Rapid Guideline: Managing the Long-Term Effects of COVID-19. https://www.nice.org.uk/guidance/ng188/resources/covid19-rapid-guideline-managing-the-longterm-effects-of-covid19-pdf-51035515742.

[8] Chen C., Haupert S.R., Zimmermann L., Shi X., Fritsche L.G., Mukherjee B. (2022). Global Prevalence of Post-Coronavirus Disease 2019 (COVID-19) Condition or Long COVID: A Meta-Analysis and Systematic Review. J. Infect. Dis..

[9] Mahase E. (2020). Long Covid Could Be Four Different Syndromes, Review Suggests. BMJ.

[10] Buonsenso D., Piazza M., Boner A.L., Bellanti J.A. (2022). Long COVID: A Proposed Hypothesis-Driven Model of Viral Persistence for the Pathophysiology of the Syndrome. Allergy Asthma Proc..

[11] Queiroz M.A.F., Neves P.F.M.D., Lima S.S., Lopes J.D.C., Torres M.K.D.S., Vallinoto I.M.V.C., Bichara C.D.A., Santos E.F.D., de Brito M.T.F.M., da Silva A.L.S. (2022). Cytokine Profiles Associated with Acute COVID-19 and Long COVID-19 Syndrome. Front Cell Infect. Microbiol..

[12] Fernández-de-las-Peñas C., Arendt-Nielsen L., Díaz-Gil G., Gil-Crujera A., Gómez-Sánchez S.M., Ambite-Quesada S., Palomar-Gallego M.A., Pellicer-Valero O.J., Giordano R. (2022). ACE1 Rs1799752 Polymorphism Is Not Associated with Long-COVID Symptomatology in Previously Hospitalized COVID-19 Survivors. J. Infect..

[13] Health Organization W. (2021). Guideline Clinical Management of COVID-19 Patients: Living Guideline, 18 November 2021. https://www.who.int/publications/i/item/WHO-2019-nCoV-clinical-2021-2.

[14] Little J., Higgins J.P.T., Ioannidis J.P.A., Moher D., Gagnon F., von Elm E., Khoury M.J., Cohen B., Davey-Smith G., Grimshaw J. (2009). STrengthening the REporting of Genetic Association Studies (STREGA)-an Extension of the Strobe Statement. PLoS Med..

[15] Amraei R., Yin W., Napoleon M.A., Suder E.L., Berrigan J., Zhao Q., Olejnik J., Chandler K.B., Xia C., Feldman J. (2021). CD209L/L-SIGN and CD209/DC-SIGN Act as Receptors for SARS-CoV-2. ACS Cent. Sci..

[16] Tso H.W., Ip W.K., Chong W.P., Tam C.M., Chiang A.K.S., Lau Y.L. (2005). Association of Interferon Gamma and Interleukin 10 Genes with Tuberculosis in Hong Kong Chinese. Genes Immun..

[17] Conde S.R.S., Feitosa R.N.M., Freitas F.B., Hermes R.B., Demachki S., Araújo M.T.F., Soares M.C.P., Ishak R., Vallinoto A.C.R. (2013). Association of Cytokine Gene Polymorphisms and Serum Concentrations with the Outcome of Chronic Hepatitis, B. Cytokine.

[18] Medina T.S., Costa S.P., Oliveira M.D., Ventura A.M., Souza J.M., Gomes T.F., Vallinoto A.C.R., Póvoa M.M., Silva J.S., Cunha M.G. (2011). Increased Interleukin-10 and Interferon-Levels in Plasmodium Vivax Malaria Suggest a Reciprocal Regulation Which Is Not Altered by IL-10 Gene Promoter Polymorphism. Malar. J..

[19] Santiago A.M., da Silva Graça Amoras E., Queiroz M.A.F., da Silva Conde S.R.S., Cayres-Vallinoto I.M.V., Ishak R., Vallinoto A.C.R. (2021). TNFA -308G>A and IL10 -1082A>G Polymorphisms Seem to Be Predictive Biomarkers of Chronic HCV Infection. BMC Infect. Dis..

[20] Barbosa H.P.M., Martins L.C., dos Santos S.E.B., Demachki S., Assumpção M.B., Aragão C.D., de Oliveira Corvelo T.C. (2009). Interleukin-1 and TNF-α Polymorphisms and Helicobacter Pylori in a Brazilian Amazon Population. World J. Gastroenterol..

[21] de Brito W.B., Queiroz M.A.F., da Silva Graça Amoras E., Lima S.S., da Silva Conde S.R.S., dos Santos E.J.M., Cayres-Vallinoto I.M.V., Ishak R., Vallinoto A.C.R. (2020). The TGFB1 -509C/T Polymorphism and Elevated TGF-Β1 Levels Are Associated with Chronic Hepatitis C and Cirrhosis. Immunobiology.

[22] Keiko Nascimento Yoshioka F., Góes Araújo A., Haydee Tavella M., Guerreiro Hamoy I., Farias Guerreiro J. (2006). Prevalence of Hereditary Risk Factors for Thrombophilia in Belém, Brazilian Amazon. Genet. Mol. Biol..

[23] Queiroz M.A.F., Santiago A.M., Moura T.C.F., Amoras E.D.S.G., Conde S.R.S.D.S., Cayres-Vallinoto I.M.V., Ishak R., Vallinoto A.C.R. (2022). The IL6-174G/C Polymorphism Associated with High Levels of IL-6 Contributes to HCV Infection, but Is Not Related to HBV Infection, in the Amazon Region of Brazil. Viruses.

[24] Oliveira L.F.D., Lima C.P.S.D., Azevedo R.D.S.S., Mendonça D.S.F.D., Rodrigues S.G., Carvalho V.L., Pinto E.V., Maia A.L., Maia M.H.T., Vasconcelos J.M. (2014). Polymorphism of DC-SIGN (CD209) Promoter in Association with Clinical Symptoms of Dengue Fever. Viral Immunol..

[25] Bai F., Tomasoni D., Falcinella C., Barbanotti D., Castoldi R., Mulè G., Augello M., Mondatore D., Allegrini M., Cona A. (2022). Female Gender Is Associated with Long COVID Syndrome: A Prospective Cohort Study. Clin. Microbiol. Infect..

[26] Falchi A., Fernández-De-Las-Peñas C., Martín-Guerrero J.D., Pellicer-Valero Ó.J., Navarro-Pardo E., Gómez-Mayordomo V., Cuadrado M.L., Arias-Navalón J.A., Cigarán-Méndez M., Hernández-Barrera V. (2022). Female Sex Is a Risk Factor Associated with Long-Term Post-COVID Related-Symptoms but Not with COVID-19 Symptoms: The LONG-COVID-EXP-CM Multicenter Study. J. Clin. Med..

[27] Riccardo M., Inciardi, Alvin C. (2022). Editorial Commentary: Long COVID-19: A Tangled Web of Lungs, Heart, Mind, and Gender. Trends Cardiovasc. Med..

[28] Sylvester S.V., Rusu R., Chan B., Bellows M., O’Keefe C., Nicholson S. (2022). Sex Differences in Sequelae from COVID-19 Infection and in Long COVID Syndrome: A Review. Curr. Med. Res. Opin..

[29] Subramanian A., Nirantharakumar K., Hughes S., Myles P., Williams T., Gokhale K.M., Taverner T., Chandan J.S., Brown K., Simms-Williams N. (2022). Symptoms and Risk Factors for Long COVID in Non-Hospitalized Adults. Nat. Med..

[30] Torjesen I. (2021). COVID-19: Middle Aged Women Face Greater Risk of Debilitating Long Term Symptoms. BMJ.

[31] Xie Y., Xu E., Bowe B., Al-Aly Z. (2022). Long-Term Cardiovascular Outcomes of COVID-19. Nat. Med..

[32] Liew S.C., Gupta E.D. (2015). Methylenetetrahydrofolate Reductase (MTHFR) C677T Polymorphism: Epidemiology, Metabolism and the Associated Diseases. Eur J. Med. Genet..

[33] Meadows D.N., Pyzik M., Wu Q., Torre S., Gros P., Vidal S.M., Rozen R. (2014). Increased Resistance to Malaria in Mice with Methylenetetrahydrofolate Reductase (Mthfr) Deficiency Suggests a Mechanism for Selection of the MTHFR 677C>T (c.665C>T) Variant. Hum. Mutat..

[34] Vickers T.J., Orsomando G., de La Garza R.D., Scott D.A., Kang S.O., Hanson A.D., Beverley S.M. (2006). Biochemical and Genetic Analysis of Methylenetetrahydrofolate Reductase in Leishmania Metabolism and Virulence. J. Biol. Chem..

[35] Hajiesmaeil M., Tafvizi F., Sarmadi S. (2016). The Effect of Methylenetetrahydrofolate Reductase Polymorphisms on Susceptibility to Human Papilloma Virus Infection and Cervical Cancer. Infect. Genet. Evol..

[36] Fodil-Cornu N., Kozij N., Wu Q., Rozen R., Vidal S.M. (2009). Methylenetetrahydrofolate Reductase (MTHFR) Deficiency Enhances Resistance against Cytomegalovirus Infection. Genes Immun..

[37] Baba H., Bouqdayr M., Saih A., Bensghir R., Ouladlahsen A., Sodqi M., Marih L., Zaidane I., Kettani A., Abidi O. (2023). Association between Methylene-Tetrahydrofolate Reductase C677T Polymorphism and Human Immunodeficiency Virus Type 1 Infection in Morocco. Lab. Med..

[38] Karakus N., Duygu F., Rustemoglu A., Yigit S. (2022). Methylene-Tetrahydrofolate Reductase Gene C677T and A1298C Polymorphisms as a Risk Factor for Crimean-Congo Hemorrhagic Fever. Nucleosides Nucleotides Nucleic Acids.

[39] Bronowicki J.P., Abdelmouttaleb I., Peyrin-Biroulet L., Venard V., Khiri H., Chabi N., Amouzou E.K., Barraud H., Halfon P., Sanni A. (2008). Methylenetetrahydrofolate Reductase 677 T Allele Protects against Persistent HBV Infection in West Africa. J. Hepatol..

[40] Ponti G., Pastorino L., Manfredini M., Ozben T., Oliva G., Kaleci S., Iannella R., Tomasi A. (2021). COVID-19 Spreading across World Correlates with C677T Allele of the Methylenetetrahydrofolate Reductase (MTHFR) Gene Prevalence. J. Clin. Lab. Anal..

[41] Moreno-Pérez O., Merino E., Leon-Ramirez J.M., Andres M., Ramos J.M., Arenas-Jiménez J., Asensio S., Sanchez R., Ruiz-Torregrosa P., Galan I. (2021). Post-Acute COVID-19 Syndrome. Incidence and Risk Factors: A Mediterranean Cohort Study. J. Infect..

[42] Yang W., Ding X., Deng J., Lu Y., Matsuda Z., Thiel A., Chen J., Deng H., Qin Z. (2011). Interferon-Gamma Negatively Regulates Th17-Mediated Immunopathology during Mouse Hepatitis Virus Infection. J. Mol. Med..

[43] Zielinski C.E., Mele F., Aschenbrenner D., Jarrossay D., Ronchi F., Gattorno M., Monticelli S., Lanzavecchia A., Sallusto F. (2012). Pathogen-Induced Human T H17 Cells Produce IFN-γ or IL-10 and Are Regulated by IL-1β. Nature.

[44] Kak G., Raza M., Tiwari B.K. (2018). Interferon-Gamma (IFN-γ): Exploring Its Implications in Infectious Diseases. Biomol. Concepts.

[45] Pirault J., Polyzos K.A., Petri M.H., Ketelhuth D.F.J., Bäck M., Hansson G.K. (2017). The Inflammatory Cytokine Interferon-Gamma Inhibits Sortilin-1 Expression in Hepatocytes via the JAK/STAT Pathway. Eur. J. Immunol..

[46] Mitok K.A., Keller M.P., Attie A.D. (2022). Sorting through the Extensive and Confusing Roles of Sortilin in Metabolic Disease. J. Lipid. Res..

[47] Queiroz M.A.F., Azevedo V.N., Amoras E.D.S.G., Moura T.C.F., Guimarães Ishak M.D.O., Ishak R., Vallinoto A.C.R., Martins Feitosa R.N. (2018). IFNG +874A/T Polymorphism among Asymptomatic HTLV-1-Infected Individuals Is Potentially Related to a Worse Prognosis. Front. Microbiol..

[48] Cordeiro P.A.S., Assone T., Prates G., Tedeschi M.R.M., Fonseca L.A.M., Casseb J. (2022). The Role of IFN-γ Production during Retroviral Infections: An Important Cytokine Involved in Chronic Inflammation and Pathogenesis. Rev. Inst. Med. Trop. Sao Paulo.

[49] Sun P., García J., Comach G., Vahey M.T., Wang Z., Forshey B.M., Morrison A.C., Sierra G., Bazan I., Rocha C. (2013). Sequential Waves of Gene Expression in Patients with Clinically Defined Dengue Illnesses Reveal Subtle Disease Phases and Predict Disease Severity. PLoS Negl. Trop. Dis..

