# Supplementary material for: Thrombophilia and Immune-Related Genetic Markers in Long COVID
Source: Viruses. 2023 Mar 30;15(4):885. doi: 10.3390/v15040885 (PMC10143911; doi:10.3390/v15040885)
Supplement: Supplementary file 1 [file viruses-15-00885-s001.zip › viruses-2296122-supplementary.pdf]

**Supplementary table S1**. Genotypic and allelic frequencies of genetic markers in relation to acute COVID-19 (NLC), Long COVID-19 patients (LC) and in Belém (BEL; prior the pandemics).

| **Gene** | Genotypes and Allelles | Frequency (%) | | | |
|---|---|---|---|---|---|
| | | Control (BEL) | NLC (Mild or asymptomatic) | LC (Mild ≥ 3m) | ***P**** |
| **TNFA** | | n (497) | n (79) | n (199) | 0.0808 |
| | **GG** | 390 (78.5%) | 67 (85%) | 183 (92%) | |
| | **AG** | 96 (19.3%) | 12 (15%) | 15 (7.5%) | |
| | **AA** | 11 (2.2%) | 0 | 1 (0.5%) | |
| | **A** | 12% | 7% | 4% | |
| **IFNG** | | n (398) | n (79) | n (199) | **0.0332*** |
| | **AA** | 226 (56.7%) | 36 (45.5%) | 119 (60%) | |
| | **AT** | 147 (37%) | 30 (38%) | 68 (34%) | |
| | **TT** | 25 (6.3%) | 13 (16.5%) | 12 (6%) | |
| | **T** | 25% | 35% | 23% | |
| **IL6** | | n (300) | n (79) | n (198) | 0.4081 |
| | **GG** | 207 (69%) | 47 (59.5%) | 129 (65%) | |
| | **CG** | 85 (28%) | 28 (35.5%) | 62 (31%) | |
| | **CC** | 8 (3%) | 4 (5%) | 7 (4%) | |
| | **G** | 83% | 77% | 80% | |
| **IL6R** | | | n (79) | n (199) | 0.4625 |
| | **AA** | ** | 31 (39%) | 59 (30%) | |
| | **AC** | ** | 33 (42%) | 100 (50%) | |
| | **CC** | ** | 15 (19%) | 40 (20%) | |
| | **C** | | 40% | 45% | |
| **CD209** | | n (72) | n (79) | n (199) | 0.0604 |
| | **AA** | 50 (69.4%) | 48 (61%) | 145 (73%) | |
| | **AG** | 19 (26.4%) | 28 (35%) | 49 (25%) | |
| | **GG** | 3 (4.2%) | 3 (4%) | 5 (2%) | |
| | **G** | 17% | 21% | 15% | |

| **Gene** | Genotypes and Allelles | Frequency (%) | | | |
|---|---|---|---|---|---|
| | | Control (BEL) | NLC (Mild or asymptomatic) | LC (Mild ≥ 3m) | ***P**** |
| **CIITA** | | | n (79) | n (199) | 1.0000 |
| | **AA** | ** | 18 (23%) | 39 (19%) | |
| | **AG** | ** | 32 (40%) | 99 (50%) | |
| | **GG** | ** | 29 (37%) | 61 (31%) | |
| | **A** | | 43% | 44% | |
| **DPA1** | | | n (79) | n (199) | 0.7893 |
| | **AA** | ** | 37 (47%) | 88 (44%) | |
| | **AG** | ** | 36 (45%) | 91 (46%) | |
| | **GG** | ** | 6 (8%) | 20 (10%) | |
| | **A** | | 70% | 67% | |
| **DPB1** | | | n (79) | n (199) | 0.5807 |
| | **AA** | ** | 31 (39%) | 70 (35%) | |
| | **AG** | ** | 41 (52%) | 100 (50%) | |
| | **GG** | ** | 7 (9%) | 29 (15%) | |
| | **G** | | 35% | 40% | |
| **MTHFR** | | n (478) | n (79) | n (199) | **0.0450*** |
| | **CC** | 201 (42%) | 28 (35%) | 98 (49%) | |
| | **CT** | 226 (47%) | 40 (51%) | 80 (40%) | |
| | **TT** | 51 (11%) | 11 (14%) | 21 (11%) | |
| | **T** | 34% | 39% | 31% | |
| **FV** | | n (127) | n (79) | n (198) | 0.3218 |
| | **GG** | 123 (97%) | 77 (97.5%) | 196 (99%) | |
| | **AG** | 4 (3%) | 2 (2.5%) | 2 (1%) | |
| | **AA** | 0 | 0 | 0 | |
| | **A** | 1.6% | 1.2% | 0.5% | |

SNPs genotype and allele frequencies in the population of Belém could be obtained from studies conducted before the COVID-19 pandemics: *TNFA* and *IFNG* [17–21], *MTHFR* and *F5* [22], *IL6* [23], *CD209* [24]. *Analysis between NLC and LC groups (Fisher's exact test). ** No date available in literature.
